# What Makes Indian Management Students Thrive? Role of Decision-Making Discretion, Broad Information Sharing, and Climate of Trust

**DOI:** 10.3389/fpsyg.2022.795262

**Published:** 2022-03-09

**Authors:** Raina Chhajer, Smita Chaudhry

**Affiliations:** ^1^Humanities and Social Sciences Area, Indian Institute of Management Indore, Indore, India; ^2^Department of Human Resources, FLAME School of Business, FLAME University, Pune, India

**Keywords:** thriving, self-determination theory, autonomy, competence, decision-making discretion, broad information sharing, climate of trust

## Abstract

Thriving is a psychological state in which individuals experience a sense of vitality and a sense of learning. Thriving come from relational connections with others, and is deeply rooted in social systems. Theoretical literature suggests that thriving occurs in the presence of decision-making discretion, broad information sharing, and a climate of trust. However, no study has investigated these environmental factors empirically. Using a multiple-studies approach, we (a) established valid and reliable scale for each of these environmental factors using experimental vignettes, (b) confirmed the association of decision-making discretion, broad information sharing and climate of trust with thriving, and (c) identified the role of self-determination theory in determining these relationships. Our analysis was based on data collected from 512 Indian management students across five studies. The results indicate significant difference in thriving for high vs. low level for decision-making discretion, broad information sharing and climate of trust. The relationship of these environmental factors with thriving is mainly due to the fulfillment of the need for competence. Competence partially mediates the relationship of decision-making discretion with thriving, and fully mediates the relationship of broad information sharing and climate of trust with thriving. Autonomy, although positively related with the environmental factors, does not lead to thriving. Practical implications, limitations and research avenues are discussed.

## Introduction

Happiness of employees is an aspiration for an organization that believes in its contribution to organizational performance. Happy employees can positively contribute to performance in many ways. Happiness leads to altruistic behavior, good health due to higher immunity, and effective management of stress (Lyubomirsky et al., 2005; De Neve et al., 2013; Layous and Lyubomirsky, 2014). These positive outcomes reduce absenteeism, promote bonhomie at the workplace and increase engagement. Consequently, organization benefits from higher productivity, collaboration, organizational citizenship behavior and performance. Thriving is one of the aspects of happiness that may be experienced by employees. It is a psychological state in which individuals feel that they are growing, developing, and are energized (Porath et al., 2012). Thriving is associated with a multitude of positive organizational outcomes such as occupational performance, low burnout or stress, job satisfaction, and organizational commitment (Spreitzer and Porath, 2012, 2014; Spreitzer et al., 2012; Parker et al., 2013; Gerbasi et al., 2015; Kleine et al., 2019).

Spreitzer et al. (2005) has used self-determination theory to conceptually identify the measures organizations can take to enable an environment for thriving for employees. Self-determination theory suggests that individuals would perform well professionally and achieve personal well-being in environments that help satisfy their three fundamental psychological needs: need of autonomy, competence and relatedness. Arguing through this theory, Spreitzer et al. (2005) has postulated that decision-making discretion, broad information sharing and climate of trust are the aspects of organizational environment that can provide psychological satisfaction leading to thriving in employees. However, there is no existing research that has empirically tested the relationship of these environmental factors with thriving. In this paper, we examine (a) the relationship of decision-making discretion, broad information sharing and climate of trust with thriving, and (b) the role of self-determination theory in determining this relationship.

The paper provides the conceptual background of thriving and self-determination theory, and the arguments concerning the hypothesized relationships. It presents a multiple studies approach using a combination of experimental and field study methods. The first study is experimental. It develops and tests scales for decision-making discretion, broad information sharing and climate of trust. For this purpose, new items for these three constructs are defined based on theoretical literature, and tested for convergent and discriminant validity and reliability. Experimental vignettes are also defined for high and low situations for the environmental factors, and are verified through *t*-tests on the developed scales. The second study series is experimental. It investigates the relationship of decision-making discretion, broad information sharing and climate of trust with thriving. For this purpose, the experimental vignettes and scales developed in Study 1 are used along with thriving scale, and the significance of the differential impact of low and high levels of these environmental factors on thriving, is verified using *t*-tests. The third study is a field study. It explores the direct relationship of these factors with thriving, and the mediating role of constructs of self-determination theory (autonomy, competence and relatedness). All the hypotheses are tested in this study using hierarchical regression analysis. Based on the findings of this study, we discuss the theoretical contribution, practical implication and research directions.

### Thriving

Thriving is a psychological state where individuals experience progress and heightened momentum at their workplace (Carmeli and Spreitzer, 2009, p. 169). It is also associated with an enhancement in short-term functioning of individuals and their long-term adaptability at work (Spreitzer et al., 2005). Thriving is perceived as a psychological state rather than a trait. Therefore, changes in thriving are seen to be extremely fluid and situationally dependent on the individuals’ environment. This makes it a challenging equilibrium to maintain. Thriving is indicated by a blend of two key factors: vitality and learning.

Vitality is the positive feeling of having energy and feeling alive at work (Nix et al., 1999). It can also be described as the energy available to an individual, either directly or indirectly from fulfilling fundamental psychological needs (Ryan and Deci, 2000). Vitality encompasses the emotive dimension of thriving. Learning is defined in terms of acquiring and applying relevant knowledge and skills at work to build proficiency (Edmondson, 1999). It has been known to have positive effects on performance (Colquitt et al., 2000). Learning signifies the cognitive dimension of thriving.

### Self-Determination Theory

Self-determination theory (SDT) (Ryan and Deci, 2000) posits that individuals are motivated and exhibit well-being, in organizational structures to the extent that they experience psychological satisfaction within those structures. The theory explains the structures that are supportive or detrimental toward these tendencies.

The theory also propounds that humans are inherently aligned toward actualizing their capabilities, through processes such as intrinsic motivation, social internalization and integration, and connecting with others (Vansteenkiste and Ryan, 2013). Intrinsic motivation is defined as an inherent inclination toward assimilation and exploration that helps individuals in their cognitive and social development. It yields greater vitality and improved conceptual learning (Deci and Ryan, 2008). Individuals, who are able to fully integrate and internalize social norms and guidelines, enact them with more effectiveness (Ryan and Connell, 1989).

Self-determination theory considers psychological needs to be innate and fundamental requisites, similar to biological needs (Ryan and Deci, 2000). The term “needs” has been used most commonly to refer to a person’s conscious wants, desires, or motives (Baard et al., 2004). Consequently, psychological need satisfaction is derived from a need to meet psychological deficit. It is regarded as the primary nutriment for individuals’ optimal functioning, psychological growth and well-being (Ryan and Deci, 2000).

The theory incorporates three dimensions corresponding to three fundamental needs that are prerequisites to psychological well-being: need for autonomy, need for competence and need for relatedness. According to Spreitzer and Porath (2012), these three dimensions explain variance in thriving, and predict affective and cognitive dimensions of thriving.

#### Need for Autonomy

Autonomy represents individuals’ innate inclination to experience volition and a sense of choice and freedom when carrying out an activity (Ryan and Deci, 2000). It requires the feeling that one is the initiator of one’s actions (Deci, 1975). Notably, both individualistic and collectivistic modes of functioning can happen volitionally.

When an individual’s behavior is autonomous in nature, it is minimally self-depleting, and creates less conflict. It also helps her/him perform well in activities requiring self-control (Muraven et al., 2008). Studies have shown that performance-contingent rewards administered in an autonomy-supportive interpersonal climate, as opposed to a controlling interpersonal climate, result in higher intrinsic motivation (Ryan et al., 1983). Autonomous forms of motivation maximize heuristic performance and commitment.

#### Need for Competence

The need for competence is interpreted as an individual’s inherent desire to feel effective in the interactions with her/his surroundings (Ryan and Deci, 2000). Internalization of extrinsically motivated activities is also a function of perceived competence. Individuals adopt activities that relevant social groups value when they feel efficacious with respect to those activities.

Competence allows individuals to feel valued, and encourages them to take risks to accomplish the tasks at hand. It further motivates them to experiment with new and more efficient methods to solve mundane problems. Competence is prominently displayed in individuals’ propensity to explore and manipulate the environment, and to engage in tasks that test and extend their skills. It promotes success at challenging tasks, and in attaining desired outcomes.

#### Need for Relatedness

The need for relatedness is defined as an individual’s inherent propensity to feel connected to others, feel loved and cared for Baumeister and Leary (1995). Relatedness calls for a sense of mutual respect, caring and reliance amongst individuals.

The need for relatedness is satisfied when individuals sense a feeling of communion, and develop intimate and lasting relationships with others (Ryan and Deci, 2000). Relatedness furthers their need for growth as they adapt and evolve by learning from experience of their counterparts (Ryan and Grolnick, 1986). It enhances intrinsic motivation, and strengthens feelings of trust between individuals. Taking decisions as a community not only affects a larger system, but also encourages them to relate to each other. Further, it facilitates internalization of required behaviors (Ryan et al., 1994). Individuals tend to imbibe values, perspectives and behavior that are considered socially acceptable.

### Enablers of Thriving

Self-determination theory explains the psychological basis of individual behavior, to pursue conditions that promote growth and progress (Ryan and Deci, 2000). Using this theoretical lens, Spreitzer et al. (2005) have provided a conceptual understanding of the influence of environmental conditions on thriving. They have identified three environmental factors that may influence thriving, namely (a) decision-making discretion, (b) broad information sharing and (c) climate of trust. We hereby elaborate on the environmental factors, and argue the role of the different dimensions of SDT in determining the impact of these factors on thriving.

#### Decision-Making Discretion

Decision-making discretion (DMD) refers to an organizational environment in which individuals have authority to exercise choice on what tasks to execute and how to execute them (Ryan and Deci, 2000). It provides them an opportunity to drive their own decisions, and feel more in control of their work without any external regulation.

Decision-making discretion can allow individuals to have influence over strategic, administrative and operating outcomes at the workplace (Ryan and Deci, 2000). People in this environment are more likely to proactively and persistently apply their skills to their tasks (Bandura, 1988). Moreover, DMD can assist individuals in forging new skill-sets, making them more comfortable in taking risks, and exploring new found opportunities (Spreitzer, 1995). It can also make them more focused about seeking out new directions for performing their tasks (Amabile, 1993), and accomplishing their job in the most efficient manner possible.

Studies have found that offering choice and encouraging self-initiation in managers, build their occupational satisfaction. Further, choice enhances intrinsic motivation and, facilitates internalization of requisite behaviors (Zuckerman et al., 1978). Besides, upskilling supervisors, so that they can maximize opportunities for their managers to take initiative, improves the managers’ attitudes toward their task (Deci et al., 1989).

Therefore, it is expected that DMD would develop the capability and energy in individuals to demonstrate work behaviors that enable them to perform well in their organization. We propose,

*H1: Decision-making discretion is positively related to thriving.*

##### Role of Autonomy

Exposure to work environments that allow discretion to make decisions, defines the degree to which individuals feel that their actions are driven by themselves rather than by external factors. This drives their sense of autonomy. DMD may manifest in terms of taking individual perspectives into account, promoting greater choice, and encouraging self-initiation (Gagné and Deci, 2005). All these factors satisfy the need for autonomy. When individuals are externally regulated, their need for autonomy is not met (Ryan and Deci, 2000). For example, employees who are forced to meet specific deadlines experience little volition in executing the assigned tasks.

Liberty in decision-making provides individuals the opportunity to accomplish their tasks the way they desire. Autonomy felt due to availability of choice, and opportunities for self-direction, enhance their intrinsic motivation (Deci and Ryan, 2008). This can consequently lead to thriving. Therefore, we propose:

*H1a: Autonomy fully mediates the relationship between decision-making discretion and thriving.*

##### Role of Competence

Individuals have an inherent need for competence. Satisfaction of this need is one of the primary forces of their motivation (Mouratidis et al., 2008). Individuals consciously undertake activities that allow them experience, and enhance their competence. When they have the freedom to make their own decisions, they assume tasks that help them harness their skills more efficiently. This increases their competence, as well as confidence in it. Studies have found that support from superiors for self-driven decisions leads to greater satisfaction of competence (Baard et al., 2004).

Challenging activities that allow individuals to showcase competence characteristics are intrinsically motivating (Danner and Lonky, 1981). Intrinsic motivation can help individuals thrive in the organization. Therefore, DMD can bring about thriving by making individuals more competent. We propose:

*H1b: Competence fully mediates the relationship between decision-making discretion and thriving.*

##### Role of Relatedness

Being part of organizational decision-making motivates individuals to relate heedfully by promoting a sense of connectedness with others (Ryan and Deci, 2000). When they have the freedom to decide what actions to take and how to execute them, individuals are also more likely to have more opportunities and openness for reaching out to their peers. They are also more likely to create positive feedback loops that help them learn new techniques.

Thus, environments characterized by a sense of secure relatedness can enable individuals to thrive (La Guardia et al., 2000). Ryan and Grolnick (1986) have shown that students, whose teachers are warm and caring, experience greater intrinsic motivation. According to research by Wall et al. (1986), structuring workflows to allow interdependence among employees and identification with workgroups, and showing concern and respect for each employee, had a positive effect on work outcomes. There is also evidence that effective workgroups can facilitate internalization of extrinsic motivation and augment thriving. James and Greenberg (1989) found that identifying with a group, which facilitates internalization of group values, led to enhanced performance. Thus, we propose:

*H1c: Relatedness fully mediates the relationship between decision-making discretion and thriving.*

#### Broad Information Sharing

Broad information sharing (BIS) refers to an organizational environment in which information is communicated widely throughout the organization (Spreitzer, 1995). Sharing information is central to “open book management,” a management methodology that advocates transparency of the organization in all its activities. Organizational leaders may provide information to their followers on aspects like opportunities to develop new skills, and feedback on their task effectiveness (Walumbwa et al., 2010). Other types of information can be organization’s vision or performance and product/service quality feedback.

Literature has discussed the benefits of certain aspects of BIS environment in an organization. Information sharing in the form of feedback resolves feelings of uncertainty in individuals. It allows them to accurately appraise themselves and evaluate their progress. It also enables them to maximize the use of their time toward personal growth and improvement (Ashford and Cummings, 1983). Feedback helps aim work-affiliated activities toward desired personal and organizational goals (Locke and Latham, 1990). Regular and adequate feedback also creates an optimal learning environment for an individual, and increases affective outcomes (Vroom, 1964). Positive feedback enhances intrinsic motivation, and facilitates internalization of requisite behaviors (Deci, 1975). It also improves individuals’ attitudes toward their task (Deci et al., 1989).

Therefore, BIS helps individuals understand the meaning and purpose of their work better, and assists them in conceptualizing how they can contribute in an appropriate manner. This can enable them to thrive and perform their job effectively. Thus, we propose:

*H2: Broad information sharing is positively related to thriving.*

##### Role of Autonomy

Broad information sharing gives individuals access to general organizational knowledge and specific information about their current performance, personal progress on goals and objectives, and relative importance of various goals to personal progress (Ashford and Cummings, 1983). With broad information access, individuals have the ability to expand their understanding of how the organizational structure functions, which can promote their feeling of autonomy (Weick and Sutcliffe, 2001).

Autonomy experienced due to BIS can equip individuals with the energy and learning opportunities, to perform assigned work well, and produce positive work outcomes. This is because they can tend to their tasks without facing hurdles and delays. Deci et al. (1989) found that providing pertinent information to individuals in a non-controlling manner was associated with higher occupational satisfaction. This is likely to lead to thriving. Thus, we propose:

*H2a: Autonomy mediates the relationship between broad information sharing and thriving.*

##### Role of Competence

A BIS environment increases the ability to swiftly decipher problems as they come to light, and coordinate actions to solve the problems. The increased capacity to respond effectively in unfamiliar situations acts as a stimulus for exploring, experimenting and learning new behaviors (Bunderson and Sutcliffe, 2002). Studies have found that information sharing, in the form of positive feedback, satisfies individuals’ inborn need for competence (Mouratidis et al., 2008). Environments that provide adequate feedback are regarded as a central factor for competence fulfillment (Deci and Ryan, 2008).

According to existing literature, an extraneous event that increases an individual’s feeling of competence (for example, positive feedback), also enhances her/his intrinsic motivation (Arnold, 1976). The individual feels more responsible for successful performance. This would lead to thriving. Thus, we propose:

*H2b: Competence mediates the relationship between broad information sharing and thriving.*

##### Role of Relatedness

Broad information sharing creates an exploratory environment where individuals are ready to reach out to each other for exchange of information. As they interact with others, they gain a deeper understanding about the intricacies of their work. As individuals comprehend how to recombine their existing knowledge in new ways to solve problems, they also realize the fitment of their work into the larger scheme of things. They are able to respond to suboptimal solutions, and increase their understanding of the working of the organization (Weick and Sutcliffe, 2001).

Existing studies have found that individuals in high-quality interpersonal relationships exchange more information and ideas, value one another, and provide a climate in which one feels safe to perform (Edmondson et al., 2004). Getting insight into the big picture enables individuals to shift focus to larger organizational contributions instead of focusing only on narrow tasks, thus enabling them to thrive. Therefore, we propose:

*H2c: Relatedness mediates the relationship between broad information sharing and thriving*.

#### Climate of Trust

Climate of trust (COT) refers to an organizational environment characterized by established and dominant behaviors that demonstrate inter-personal trust. The behaviors may involve acknowledging and considering perspectives of individuals while taking decisions, and including them in the decision-making process (Lawler, 1992). They may also include offering choice, and encouraging self-initiation (Deci et al., 1989), thus leading to higher job satisfaction and self-regulation.

Climate of trust creates situations favorable for creative engagement and thriving. When individuals have trust, they are more willing to take risks (Mayer et al., 1995; Edmondson, 1999). Also, COT facilitates experimentation with new behaviors, since individuals feel safe enough to explore (Spreitzer, 1995; Bunderson and Sutcliffe, 2002). Work environments and managerial methods, which create a climate of trust, promote basic need satisfaction, intrinsic motivation and appropriate internalization of extrinsic motivation. This in turn leads to persistence, positive work attitudes, organizational commitment, and psychological well-being. Therefore, climate of trust is critical to promote thriving in individuals. Thus, we propose:

*H3: Climate of trust is positively related to thriving.*

##### Role of Autonomy

Climate of trust encourages individuals to be open to voice their thoughts and conduct activities the way they want. Thus, it promotes freedom and autonomy to execute tasks. COT can allow autonomy to individuals in areas like goal setting, decision-making and task planning. It can create a sense of empowerment, leading to enhanced self-determination.

When individuals are situated in COT, they are likely to feel more constructive and confident of conquering challenges in their immediate surroundings (Spreitzer, 1995). Feeling of autonomy created by COT can promote internalization and integration of extrinsic motivation, causing positive outcomes. Studied have found that self-determination resulting from a sense of empowerment leads to thriving (Champy, 1995). Thus, we propose:

*H3a: Autonomy mediates the relationship between climate of trust and thriving.*

##### Role of Competence

Climate of trust encourages individuals to invest their resources in an unconstrained manner, and completely commit their effort and time in executing the assigned tasks. They know that they would get help and positive feedback from others along the way to help them perform successfully. Positive feedback enables individuals to take risks while making them feel an integral member of the organization, thus promoting their competence (Deci, 1975). Also, COT creates a supportive environment that is central for competence fulfillment (Deci and Ryan, 2008).

Competence due to positive feedback comes with a sense of responsibility for individual performance, and an appreciation about its effect on organizational performance (Fisher, 1978). Competence resulting from COT thus promotes energy and a drive toward learning, with the objective of performing well as a part of the organization. We propose:

*H3b: Competence mediates the relationship between climate of trust and thriving.*

##### Role of Relatedness

When individuals are exposed to COT, it invokes a sense of belongingness and a belief that they are valued members of the organization. Consequently, this fosters a sense of relatedness, as they feel much more understanding with regard to each other (Rhoades and Eisenberger, 2002).

The sense of relatedness created by COT allows individuals to collaborate with each other, and work together toward attaining high levels of performance. It also sparks feelings of positive emotions, leading to increased vitality and openness to learning (Fredrickson, 2001). Thus, we propose:

*H3c: Relatedness mediates the relationship between climate of trust and thriving.*

To test these hypotheses, we performed a series of studies. This was required since some constructs (DMD, BIS and COT) did not have existing scales, and empirical analysis on them was possible only after we had defined and tested scales. The first study helped to develop and test scales for DMD, BIS and COT. The second study helped to verify the results obtained from the first study, and assess how thriving differed for low and high levels of DMD, BIS and COT (and thus how these environmental factors impacted thriving). The third study helped to verify their impact on thriving, and tests all the hypotheses. The three studies were conducted on different sets of participants to prevent consistency bias.

## Study 1

The objective of Study 1 was to define and validate measures for decision-making discretion, broad information sharing and climate of trust, as they do not have pre-existing measures. To enable this process, we defined new scales and tested them through an experimental study on a sample of management students. For this purpose, we also created experimental vignettes. Experimental vignettes are small scenarios representing commonly faced real life situations (Vargas, 2008; Atzmüller and Steiner, 2010; Aguinis and Bradley, 2014). They are used in experimental studies, and are accompanied by a set of questions. Respondents are required to respond to the questions, based on their assessment of the given situation. Experimental vignette method helps to establish causal relationships. The content of the vignette is structured, and controls for factors that may confound the results (Aguinis and Bradley, 2014). It also helps to test item scales by enabling manipulation check. Besides, use of vignettes reduces scope for social desirability and acquiescence bias (Podsakoff et al., 2003).

We carried out the following steps to develop the item scales. First, we defined the items and vignettes based on the existing theoretical literature on decision-making discretion, broad information sharing and climate of trust (Spreitzer et al., 2005). For each variable, we defined two versions of the vignette: one representing a low level, and the other representing the high level. Second, we conducted an exploratory factor analysis on the items for all variables together, to confirm that the three variables were valid independent constructs. Third, we calculated the average variance extracted for each variable to confirm their convergent validity. Fourth, we compared it to the correlation between variables to confirm their discriminant validity. Fifth, we calculated the Cronbach’s Alpha of each variable to confirm reliability. Sixth, we conducted *t*-tests to check for significance of difference between high and low levels of each vignette, to confirm content validity of the variables.

### Method

#### Participants

Two hundred and forty students (*n* = 108 females; *n* = 132 males) from an Indian Institute of Management participated in the study. The mean age was 18.85 years and SD was 1.3. Participation in the study was voluntary and no compensation was provided to the students. Participants were assured that their responses would remain anonymous. After obtaining participants informed consent, the survey was administered with the help of two research assistants. The research assistants collected data over a period of time from students across campus. Average time taken to complete the survey was 10 min.

#### Procedure

Based on the description of DMD, BIS and COT in the context of thriving (Spreitzer et al., 2005), we defined items for each of these variables. We also defined two vignettes for each variable; one denoting a situation in which DMD, BIS or COT is high, and the other denoting a situation in which DMD, BIS or COT is low. A total of six vignettes were defined (Supplementary Appendix A). Thus, we created eight scenarios representing a 2 (high vs. low DMD) × 2 (high vs. low BIS) × 2 (high vs. low COT) between-subjects research design. Each scenario contained three vignettes corresponding to either a high or a low level DMD, BIS and COT. Use of vignettes ensured that the responses were standardized and not influenced by acquiescence bias.

Thirty students were allocated to each one of the eight scenarios on a random basis. Tests revealed no statistical difference in the demographics of the students assigned to the eight scenarios. Every student responded to items on DMD, BIS and COT for each scenario.

#### Measures

Measures for DMD, BIS and COT were created with five items each. Based on the given situation, the participants responded to the items on a scale ranging from 1 (*Strongly Disagree*) to 7 (*Strongly Agree*). Exploratory factor analysis (EFA) using promax rotation, and Cronbach’s Alpha (α) was used to test validity and reliability of the measures.

All items of DMD are provided in Table 1. Items include “You have the autonomy to do the assignment the way you want,” “You are free to select the real-life situation from any time era” and “You can choose how short you want to make the assignment.”

**TABLE 1:** Item scales for study 1 and study 2A, 2B and 2C.

	Preliminary studies	Study 1	Study 2A, 2B, 2C

S. No	Item scales	Factor loading	Factor loading
**A**	**Decision-making Discretion (Study 1: α = 0.71; Study 2: α = 0.75)**		
1	You have the autonomy to do the assignment the way you want to	0.76	0.86
2	You feel the freedom about how you want to do the assignment	0.74	0.84
3	You are free to select the real-life situation from any time era	0.61	0.70
4	You feel in control about what you want to do in the assignment	0.65	0.65
5	You can choose how short you want to make the assignment	0.61	0.62
**B**	**Broad Information Sharing (Study 1: α = 0.78; Study 2: α = 0.84)**		
1	You know the different sections which will constitute the assignment	0.86	0.68
2	You are aware about the layout, font and line spacing to be used for the assignment	0.84	0.66
3	You have been told about the structure and flow of the assignment	0.79	0.65
4	You knew how you would go about collecting information about your assignment	0.69	0.63
5	You have information about the format of the assignment	0.63	0.63
**C**	**Climate of Trust (Study 1: α = 0.87; Study 2: α = 0.91)**		
1	The faculty makes it comfortable to seek clarifications	0.87	0.94
2	The faculty provides a secure environment for exchanging ideas and doubts	0.82	0.85
3	You can rely on the faculty for giving honest feedback	0.81	0.78
4	The faculty values your opinion and perspectives	0.80	0.72
5	The faculty makes it possible to improve assignment quality through open discussion	0.74	0.75
**D**	**Thriving (Study 1: Not Applicable; Study 2: α = 0.78)**		
1	While doing this assignment, you would often get new insights about the course		0.81
2	You would continue to learn more, as you spend more time on this assignment		0.80
3	You would see yourself getting continuously better at the selected concepts		0.78
4	Doing this assignment will not make you learn anything (R)		0.77
5	While doing this assignment, you would develop a lot as a learner of the course		0.75
6	You would feel alive and vital while doing this assignment		0.65
7	The assignment would give you energy and spirit		0.64
8	You would not feel very energetic about doing the assignment (R)		0.64
9	Doing this assignment will make you feel alert and awake		0.60
10	While doing this assignment, you would look forward to each day you work on the assignment		0.64

*α, Cronbach’s alpha; Study 1: N = 240; Study 2: N_2A_ = 60; N_2B_ = 60; N_2C_ = 60.*

All items of BIS are provided in Table 1. Items include “You know the different sections which will constitute the assignment,” “You are aware about the layout, font and line spacing to be used for the assignment” and “You have been told about the structure and flow of the assignment.”

All items of COT are provided in Table 1. Items include “The faculty makes it comfortable to seek clarifications,” “The faculty provides a secure environment for exchanging ideas and doubts” and “You can rely on the faculty for giving honest feedback.”

### Results and Discussion

Exploratory factor analysis for items of all the variables together resulted in extraction of three factors with insignificant cross-loading of items across factors. Items for one variable loaded on one factor, and all factor loadings were more than 0.60. The average variance extracted (AVE) for each variable was more than 0.5, denoting convergent validity. Besides, the square root of AVE was greater than the correlation between the variables, denoting discriminant validity (Fornell and Larcker, 1981).

The reliability for DMD (α = 0.71), BIS (α = 0.78) and COT (α = 0.87) was acceptable. The mean statistic for DMD, BIS and COT was used to conduct *T*-tests for manipulation check of the vignettes. The results revealed that there was significant difference between the vignettes representing high and low DMD [*t* (238) = 5.74, *p* < 0.001, *d* = 0.74], high and low BIS [*t* (238) = 7.48, *p* < 0.001, *d* = 1.06], and high and low COT [*t* (238) = 7.14, *p* < 0.001, *d* = 0.92].

The results of Study 1 denoted that DMD, BIS and COT were three distinct and independent variables. The new scales developed for DMD, BIS and COT had convergent and discriminant validity, and reliability. Therefore, these scales could be considered for collecting data in other studies. Also, the vignettes, designed for high and low levels of DMD, BIS and COT, were statistically valid. The high and low levels of each variable were significantly different from each other. Therefore, we could use these vignettes in other studies for further analysis of the variables.

## Studies 2A, 2B and 2C

We followed Study 1 with three experimental studies to examine the relationship of DMD and thriving, BIS and thriving, and COT and thriving. For this purpose, we used the item scales and the vignettes created in Study 1, and collected data from another sample of management students (different from Study 1).

The objective of the studies was to test whether thriving differed for high vs. low level of DMD (Study 2A), high vs. low level of BIS (Study 2B), and high vs. low level of COT (Study 2C).

### Method

#### Participants

Sixty students from an Indian Institute of Management were randomly allocated to each study. The participants for Study 2A (*n* = 25 females; *n* = 35 males) had a mean age of 19.68 years and SD = 0.91. The participants for Study 2B (*n* = 28 females; *n* = 32 males) had a mean age of 19.51 years and SD = 0.98. The participants for Study 2C (*n* = 26 females; *n* = 34 males) had a mean age of 19.61 years and SD = 0.94. Participation in the study was voluntary and no compensation was provided to the students. Participants were assured that their responses would remain anonymous. After obtaining participants informed consent, the survey was administered with the help of two research assistants. The research assistants collected data over a period of time from students across campus. Average time taken to complete the survey was 15 min.

#### Procedure

Data were collected through questionnaires using the vignettes and the items defined in Study 1. The three studies used three different questionnaires corresponding to DMD, BIS, and COT (independent variables). The purpose of each study was to test the relationship of high vs. low level of an independent variable with thriving in a between-subjects research design. Therefore, each study used two versions of a questionnaire; one with a scenario (vignette) for high level of DMD, BIS, or COT, and the other with a scenario (vignette) for low level of DMD, BIS or COT. Use of vignettes ensured that the responses were not influenced by social desirability bias.

For each study, 30 students were assigned each version of the questionnaire on a random basis. Each student responded to items on one independent variable (DMD, BIS or COT) and thriving for one scenario. Tests revealed no statistical difference in the demographics of the students assigned to the two scenarios for each study.

#### Measures

For DMD, BIS and COT, item scales created in Study 1 were used. Measure for thriving was adapted from an established 10-item scale (Porath et al., 2012). The scale included five items each on vitality and learning (the two dimensions of thriving) with two items reverse coded. Sample items are “While doing this assignment, you would often get new insights about the course,” “You would see yourself getting continuously better at the selected concepts” and “You would feel alive and vital while doing this assignment.” The participants responded to the items on the questionnaires on a scale ranging from 1 (*Strongly Disagree*) to 7 (*Strongly Agree*).

Exploratory factor analysis indicated the two factors corresponding to the items for vitality and learning. All 10 items were retained after EFA since all had accepted factor loading and no cross loading. Thriving scale also had acceptable reliability (α = 0.78). Factor loading and Cronbach’s Alpha for all items are provided in Table 1.

### Results and Discussion

The mean statistic for the variables was used to conduct *T*-tests. Results of manipulation check showed that there was significant difference between the vignettes representing high and low DMD [*t* (58) = 3.04, *p* < 0.01, *d* = 0.78] in Study 2A, high and low BIS [*t* (58) = 2.31, *p* < 0.05, *d* = 0.58] in Study 2B, and high and low COT [*t* (58) = 3.4, *p* < 0.01, *d* = 0.88] in Study 2C. This supported manipulation of the situations.

Two-sided *t* tests were conducted to examine the relationship of DMD, BIS and COT with thriving. Results of Study 2A revealed that thriving was significantly different between high and low DMD [*t* (58) = 4.07, *p* < 0.05, *d* = 0.64]. Results of Study 2B revealed that thriving was significantly different between high and low BIS [*t* (58) = 3.98, *p* < 0.05, *d* = 0.57]. Results of Study 2C revealed that thriving was significantly different between high and low COT [*t* (58) = 4.11, *p* < 0.05, *d* = 0.83].

The results of Study 2A, 2B and 2C denoted that DMD, BIS and COT significantly related to thriving. This empirical finding confirmed existing theoretical literature (Spreitzer and Porath, 2014), and indicated that the nature of the relationships could be further investigated. Thus, we proceeded to examine these relationships in detail, along with the role of SDT, through a field study.

## Study 3

The objective of Study 3 was to investigate the relationship of DMD, BIS and COT with thriving, and the mediating role of autonomy (NAT), competency (NCM) and relatedness (NRL) (based on SDT). To enable this process, we conducted a field study on a sample of management students.

### Method

#### Participants

Ninety-two students (*n* = 34 females; *n* = 58 males) from an Indian Institute of Management participated in the study. The mean age was 28.72 years and SD = 0.64. Participation in the study was voluntary and no compensation was provided to the students. Participants were assured that their responses would remain anonymous. After obtaining participants informed consent, the survey was administered with the help of two research assistants in the classroom. Average time taken to complete the survey was 20 min.

#### Procedure

As a part of the course on “Positive Organizational Psychology,” we designed an assignment on the basis of which we could collect data. The assignment required students to collect stories of their best selves from individuals who knew them well, and analyze their own stories to distil their signature strengths. The instructor encouraged the students to invest in the assignment, provided the submission guidelines in detail and supported the students throughout. Participants responded to a questionnaire survey administered to them 2 days after submission of the assignment.

To prevent consistency bias, the survey was designed such that the participants had to provide their response to items on Thriving, followed by NAT, NCM, NRL and DMD, BIS and COT in the given order. At the end of the survey, they had to answer questions on demographics.

#### Measures

Since Study 3 was a field study, we adapted the item scales used in the experimental studies 2A, 2B and 2C, for DMD, BIS and COT and Thriving. The statements were framed in the past tense since data had to be captured regarding an actual assignment that had been conducted earlier.

Scales for NAT, NCM and NRL were adapted from existing scales (Table 2). The participants responded to the items on the questionnaires on a scale ranging from 1 (*Strongly Disagree*) to 7 (*Strongly Agree*). They also provided information on age and gender, which were used as control variables. Factor loading and Cronbach’s Alpha for all selected items are provided in Table 2.

**TABLE 2:** Item scales for study 3.

S. No	Item scales	Factor loading
**A**	**Decision-making Discretion (α = 0.79)**	
1	You had the autonomy to do the assignment the way you wanted to	0.79
2	You felt the freedom about how you wanted to do the assignment	0.75
3	You were free to select stories from any time in your life	0.72
4	You felt in control about what you wanted to do in the assignment	0.71
5	You could choose how short you wanted to make the assignment	0.61
**B**	**Broad information sharing (α = 0.80)**	
1	You knew the different sections which would constitute the assignment	0.78
2	You were aware about the layout to be used for the assignment	0.77
3	You had been told about the structure and flow of the assignment	0.73
4	You knew how you would go about collecting information about your assignment	0.72
5	You had information about the format of the assignment	0.72
**C**	**Climate of trust (α = 0.88)**	
1	The faculty made it comfortable to seek clarifications	0.86
2	The faculty provided a secure environment for exchanging ideas and doubts	0.85
3	You could rely on the faculty for giving honest feedback	0.81
4	The faculty valued your opinion and perspectives	0.80
5	The faculty made it possible to improve assignment quality through open discussion	0.80
**D**	**Autonomy (α = 0.75)**	
1	You felt that you can do the assignment being yourself	0.86
2	While doing the assignment, you did not feel constrained by the instructions provided by your faculty	0.81
3	If you could choose, you would have done your assignment differently	0.72
4	The way you were required to do the assignment was in line with how you really wanted to do it	0.68
5	You were free to do the assignment the way you thought it could best be done	0.67
**E**	**Competence (α = 0.85)**	
1	You felt that you could do the assignment exactly the way you wanted to	
2	You could master the way of doing the assignment	0.84
3	You felt competent about doing the assignment well	0.81
4	You were good at doing the assignment	0.80
5	You felt that you could accomplish even the most difficult part of the assignment	0.79
6	You knew exactly how to do the assignment	0.70
**F**	**Relatedness (α = 0.33)**	
1	In class, you felt part of the group of people who were working on the same assignment	0.84
2	You could approach your faculty with questions/doubts regarding the assignment	0.84
3	In class, you could talk with your faculty about things that really mattered to you concerning the assignment	0.82
4	You could depend on your faculty to listen to your ideas	0.81
5	Some of your classmates are close friends with whom you could discuss the assignment	0.64
**G**	**Thriving (α = 0.78)**	
1	While doing the assignment, you often got new insights about the selected concepts	0.78
2	You continued to learn more, as you spent more time on this assignment	0.73
3	You saw yourself getting continuously better at the selected concepts	0.71
4	Doing this assignment did not make you learn anything (R)	0.70
5	While doing this assignment, you developed a lot as a student of this course	0.69
6	You felt alive and vital while doing this assignment	0.63
7	The assignment gave you energy and spirit	0.69
8	You did not feel very energetic about doing the assignment (R)	0.68
9	Doing this assignment made you feel alert and awake	0.64
10	You looked forward to each day you worked on this assignment	0.63

*α, Cronbach’s alpha; R, reverse coded; N = 92.*

Measure for NAT was adapted from an established six-item scale (Broeck et al., 2010). Sample items are “You felt that you can do the assignment being yourself,” “If you could choose, you would have done your assignment differently” and “The way you were required to do the assignment was in line with how you really wanted to do it.” EFA resulted in dropping of one item due to poor factor loading. The NAT variable with the remaining five items had acceptable reliability (α = 0.75).

Measure for NCM was adapted from an established six-item scale (Broeck et al., 2010). Sample items are “You could master the way of doing the assignment,” “You felt competent about doing the assignment well” and “You knew exactly how to do the assignment.” Post EFA, all items were retained due to acceptable factor loading. The NCM variable with the selected six items had acceptable reliability (α = 0.85).

Measure for NRL was adapted from an established six-item scale (Broeck et al., 2010). Sample items are “You could approach your faculty with questions/doubts regarding the assignment,” “You could depend on your faculty to listen to your ideas” and “In class, you could talk with your faculty about things that really mattered to you concerning the assignment.” EFA resulted in dropping of one item due to poor factor loading. The NRL variable with the remaining five items had very poor reliability (α = 0.33). Therefore, we could not consider NRL for further analysis, and had to drop this variable from our model. Thus, Hypotheses 1c, 2c and 3c remained untested.

### Results and Discussion

The descriptive statistics for the variables with the retained items are provided in Table 3. We conducted a series of regression analysis using the approach suggested by Barron and Kenny (1986). Results based on standardized coefficients are as follows: a) DMD (β = 0.24, *p* < 0.05), BIS (β = 0.21, *p* < 0.05) and COT (β = 0.27, *p* < 0.01) were all significantly related to thriving; b) DMD (β = 0.48, *p* < 0.001) and COT (β = 0.31, *p* < 0.01) were significantly related to autonomy, but BIS (β = −0.03, *ns*) was not; c) all DMD (β = 0.23, *p* < 0.05), BIS (β = 0.31, *p* < 0.01) and COT (β = 0.26, *p* < 0.01) were significantly related to competence; d) competence (β = 0.56, *p* < 0.001) was significantly related to thriving, but autonomy (β = 0.16, *ns*) was not. These results suggest that all three independent variables are associated with thriving, and competence has a mediating role to play.

**TABLE 3:** Means, standard deviation and inter-correlations for study 3.

	Variables	M	S.D.	1	2	3	4	5	6	7	8
1	Gender	0.20	0.40	1.00							
2	Age	28.72	0.64	0.05	1.00						
3	Decision Making Discretion	4.57	1.06	−0.20*	0.04	1.00					
4	Broad Information Sharing	5.33	1.02	−0.01	0.04	0.45***	1.00				
5	Climate of Trust	5.37	1.09	−0.12	−0.13	0.51***	0.52***	1.00			
6	Autonomy	4.00	1.38	−0.13	−0.13	0.55***	0.29**	0.52***	1.00		
7	Competence	4.41	1.20	−0.09	−0.07	0.53***	0.58***	0.58***	0.48***	1.00	
8	Thriving	4.19	1.42	−0.04	0.09	0.59***	0.53***	0.52***	0.38**	0.70***	1.00

N = 92. *p < 0.05; **p < 0.01; and ***p < 0.001. One tailed significance values are reported.

To confirm the results obtained above, we conducted a hierarchical regression analysis containing all the variables. Hierarchical regression allows examining the effect of independent variables on the dependent variables in an incremental manner. It helps to understand the direct relationship between independent and dependent variables, as well as the impact of intervening (or mediating) variables on these direct relationships, and thus enables testing of mediation relationships. Results of the analysis are provided in Table 4. Regression consisted of three models. Model 1 included only the control variables. Model 2 included the independent variables comprising DMD, BIS and COT also. Model 3 included the mediating variables comprising NAT and NCM also.

**TABLE 4:** Hierarchical regression for thriving.

Predictor variables	Model 1	Model 2	Model 3
**Control Variables**			
Gender	0.04	0.06	0.07
Age	0.09	0.09	0.11
**Independent Variable**			
Decision Making Discretion		0.37**	0.28*
Broad Information Sharing		0.23*	0.07
Climate of Trust		0.23*	0.12
Mediating Variables			
Autonomy			0.06
Competence			0.48***
R^2^	0.01	0.47	0.58
ΔR^2^	0.01	0.46	0.12
Adjusted R^2^	0.02	0.43	0.54
F	0.31	11.28***	12.42***
ΔF	0.31	18.43***	8.58**

N = 92. *p < 0.05; **p < 0.01; and ***p < 0.001.

*All coefficients are standardized; One-tail significance values are reported.*

Results showed that the model fit increased in significance with the inclusion of independent variables [Model 2: *F* (5, 86) = 11.28, *p* < 0.001, ΔR^2^ = 0.46]. Thriving was positively related to DMD (Model 2: β = 0.37, *p* < 0.01), BIS (Model 2: β = 0.23, *p* < 0.05) and COT (Model 2: β = 0.23, *p* < 0.05). This confirmed the results obtained earlier. Thus, Hypotheses 1, 2 and 3 found support.

When autonomy and competence were included in regression, the model fit increased in significance [Model 3: *F* (7, 84) = 12.42, *p* < 0.001, ΔR^2^ = 0.12]. In particular, competence had a significant intervening role (Model 3: β = 0.48, *p* < 0.001) whereas autonomy did not (Model 3: β = −0.06, *ns*). This supported the results obtained earlier, suggesting that competence mediated the relationship of all independent variables with thriving, but autonomy did not. Thus, Hypothesis 1a, 2a and 3a did not find support, and Hypotheses 1b, 2b and 3b found support.

Table 4 also showed that inclusion of Model 3 weakened the relationship of DMD with thriving (Model 3: β = 0.28, *p* < 0.05), but made the relationship of BIS (Model 3: β = 0.07, *ns*) and COT (Model 3: β = 0.12, *ns*) with thriving completely insignificant. This indicates that competence partially mediated the relationship between DMD and thriving, and fully mediated the relationship between BIS and thriving, and COT and thriving.

To summarize, the results of Study 3 showed that DMD, BIS and COT were significantly related to thriving. The relationship for DMD, BIS and COT was mediated by competence. However, autonomy had no role to play in determining the relationship of these factors with thriving.

## General Discussion

The purpose of this research was to empirically examine the relationship of environmental factors (namely, DMD, BIS and COT) with thriving, and the role of SDT in determining this relationship. The environmental factors were based on the conceptual literature on select organizational characteristics that have been theoretically explored for their influence on thriving (Spreitzer et al., 2005). We conducted these experiments with the students at an Indian Institute of Management. These management students would be joining various organizations as future employees. Our study is indicative of the effect of environmental factors of DMD, BIS, and COT on thriving in the organizational context.

The research involved the following activities: establishing DMD, BIS and COT as three independent constructs, defining valid and reliable measure for each one of them, statistically testing their relationship with thriving, and examining the intervening role of the dimensions of SDT (namely need for autonomy, need for competence and need for relatedness). The research was carried out using five studies (Supplementary Appendix B). Study 1 involved experimental vignettes to establish the validity and reliability of the measures for DMD, BIS and COT. It confirmed them as three distinct constructs with valid and reliable scales. It also established validity of the vignettes. Study 2A, 2B and 2C used the same experimental vignettes to investigate the relationship of DMD, BIS and COT with thriving. They confirmed the significant relationship of these three factors with thriving. Study 3 involved a field survey to test the hypotheses regarding the direct role of DMD, BIS and COT, and the mediating role of autonomy, competence and relatedness, in determining thriving. The results showed that DMD, BIS and COT related to thriving as hypothesized. However, mediation was indicated only for competence. Competence partially mediated the relationship of DMD, and fully mediated the relationship of BIS and COT with thriving. However, autonomy did not have a significant intervening influence. Relatedness could not be tested due to poor reliability of items. The research indicated that a) the selected environmental factors of DMD, BIS and COT are associated with thriving; b) this association is mainly because of fulfillment of the need for competence; c) competence tends to be the only dimension of SDT that has a role to play in determining this association.

Previous studies had conceptualized environmental factors like DMD, BIS and COT in the context of SDT and thriving. However, empirical literature on these variables was absent, and measures did not exist for them. This research empirically established them as three independent constructs by defining item scales for each one of them, testing them through experimental vignettes and confirming their discriminant validity, convergent validity and reliability. By enabling these constructs with the associated measures for further research, the paper contributes to the literature on organization factors that may promote individual performance (Porath et al., 2012; Goh et al., 2021).

The paper empirically confirmed that DMD, BIS and COT related to thriving due to satisfaction of the need for competence. The results suggest that organizations can promote individual thriving by enhancing their competence, through an environment that allows discretion to make decisions, sharing of information and a climate of trust. They thus imply that organizations can prioritize certain environmental conditions, which make individuals more competent, in order to make them thrive. By empirically highlighting the critical importance of satisfaction of the need of competence (vis-à-vis autonomy) for determining individual thriving, this paper extends the literature on thriving (Spreitzer et al., 2005) and competence (Bunderson and Sutcliffe, 2002).

The results show that competence partially mediates the relationship between DMD and thriving, and fully mediates the relationship of BIS and COT with thriving. This implies that individuals, who are given the discretion to make decisions, may demonstrate thriving behaviors, because this condition also satisfies their need for competence. Further, individuals with whom broad information is shared, and who work in a climate of trust, may demonstrate thriving behaviors, just because these conditions satisfy their need for competence. The empirical findings underscore the process by which organizational factors promote individual thriving. This adds to the literature on antecedents of thriving (Porath et al., 2008).

The findings also demonstrate that the discussed organizational factors do not satisfy the need for autonomy. This suggests that autonomy has no contribution to make in determining the relationship of these factors with thriving. As the results show significance of only competence, it can be argued that SDT (with all three dimensions) does not explain the role of organizational initiatives in encouraging individual thriving. This further adds to the literature on thriving (Spreitzer et al., 2012).

### Practical Implications

Thriving plays a critical role in sustaining physical health as well as promoting positive workplace behavior (Keyes, 2002). It provides an array of positive outcomes for employers. Thriving employees are keen at taking career development initiatives and forming supportive and successful relationships with co-workers (Parker and Sprigg, 1999; Porath et al., 2008). Supervisors’ rate them as high performers. By generating new ideas, and seeking new ways of working, thriving employees exhibit more innovative work behavior (Carmeli and Spreitzer, 2009). Thriving is also linked with lower burnout and fewer physical or somatic grievances. Besides, it is found to be related to positive affect, learning goal orientation, proactive personality, and core self-evaluation (Porath et al., 2012). Therefore, it is beneficial for organizations to promote thriving both from the perspective of organizational performance, and employee satisfaction and development.

According to the findings of the paper, organizations should define norms, policies and processes to encourage employee discretion in decision-making, maintain a consistent and effective flow of important and useful information to employees, and create a climate of mutual trust across employees, hierarchical levels and departments. They can assess effectiveness of such norms, policies and processes by collecting data on employee competence on a regular basis. To ensure satisfaction of the need for competence, organizations can identify related enterprise-wide initiatives that can increase employee competence. Examples are focused and measured training programs, job rotation, job enhancement, performance feedback mechanism, and coaching and mentoring.

### Limitations

Despite the theoretical and empirical contribution of the studies, the paper also has a few limitations. We could not investigate need for relatedness because of unacceptable reliability of the measure. Compared to the reliability of other dimensions of SDT, reliability of relatedness was very low. A possible reason can be the prior inter-personal relationships of the respondents within the sample. It is likely that some respondents already related to their peers and faculty very well outside the class, and others did not. The internal classroom environment or the specific assignment did not notably influence their degree of relatedness with their peers and faculty. Another limitation of the study pertains to the relevance of our findings in other cultural contexts. We conducted these experiments with the Indian management students. These findings might be relevant to other management students in similar cultures. However, further studies might be needed to assess if these findings tend to be true for students in other contexts.

### Future Research Avenues

We conducted our empirical research on students to enable a controlled environment for defining the measures for environmental factors, creating and testing vignettes and validating the previously defined conceptual framework (Spreitzer and Porath, 2014). To confirm the findings on the environmental determinants of individual thriving, the next step would be to replicate this research across organizations and employees. This would help verify the applicability of the framework in the organizational context. Besides, this would enable comparison of the significance of different environmental factors for thriving, and of the intervening role of SDT across educational and organizational environments.

The paper has established DMD, BIS and COT as constructs, and provided valid and reliable measures for them. Future research can further explore these constructs in terms of a) their antecedents (e.g., leadership, structure and culture), and b) other consequences (e.g., organizational citizenship behavior, employee commitment, job engagement). This would facilitate a detailed understanding about the organizational characteristics that would promote a conducive environment for employee performance.

The results show that satisfaction of the need for autonomy is just one of the ways in which individual discretion to make decisions can relate to thriving. Future research can try to identify the other consequences of this discretion that bring about thriving. This might also open the doors to other theories (apart from SDT), that might explain the association of environmental factors with thriving.

## Conclusion

The purpose of this paper was to use empirical research to validate an existing conceptual framework on the relationship of select environmental factors (DMD, BIS and COT) with thriving, and the intervening role of SDT. The research was conducted in three stages through multiple studies. In the first stage, we defined vignettes and scales for the environmental factors, and collected data to establish their validity and reliability. In the second stage, we verified the relationship of these factors with thriving. In the third stage, we examined the combined association of these factors with thriving, considering their role in satisfying need for autonomy, competence and relatedness. The findings suggested partial support for the conceptual framework. The environmental factors related to individual thriving mainly due to satisfaction of the need for competence. The paper highlights certain steps organizations can take to make the workforce happy, and help them thrive. We hope that it effectively paves the way for empirical research in different organizational contexts to affirm the process by which these environmental factors are related to employee performance.

## Data Availability Statement

The raw data supporting the conclusions of this article will be made available by the authors, without undue reservation.

## Ethics Statement

Ethical review and approval was not required for the study on human participants in accordance with the local legislation and institutional requirements. The patients/participants provided their written informed consent to participate in this study.

## Author Contributions

RC: conception of the study, variables, literature review, formation of hypotheses, data collection, and interpretation. SC: research design, introduction, analysis, interpretation, and discussion. Both authors contributed to the article and approved the submitted version.

## Conflict of Interest

The authors declare that the research was conducted in the absence of any commercial or financial relationships that could be construed as a potential conflict of interest.

## Publisher’s Note

All claims expressed in this article are solely those of the authors and do not necessarily represent those of their affiliated organizations, or those of the publisher, the editors and the reviewers. Any product that may be evaluated in this article, or claim that may be made by its manufacturer, is not guaranteed or endorsed by the publisher.
