# Supplementary material for: What Makes Indian Management Students Thrive? Role of Decision-Making Discretion, Broad Information Sharing, and Climate of Trust
Source: Front Psychol. 2022 Mar 9;13:795262. doi: 10.3389/fpsyg.2022.795262 (PMC8959831; doi:10.3389/fpsyg.2022.795262)
Supplement: Supplementary file 1 [file Data_Sheet_1.docx]

**APPENDIX A**

**VIGNETTES**

**Decision Making Discretion (DMD)**

1. DMD - High

The faculty has allowed you to take your own decisions regarding certain aspects of the assignment. You can decide about the following:

- The concepts you wish to apply to a real life situation
- The period of time from where you wish to select the real life situation
- The number of pages (length) you wish to write in the document

1. DMD - Low

The faculty has specified the following with regard to the assignment. You do not have an option to deviate from these instructions

- Four out of five specific concepts (given by faculty) have to be used
- The real life situation should be recent (occurring within the last 6 months, and not before that)
- The length of the document has to be more than 10 pages

**Broad Information Sharing (BIS)**

1. BIS - High

The faculty has provided the following information to give some clarity on the assignment:

- The document has to have portrait layout, with a font of Times New Roman 12 and double spacing between lines.
- The document should have the following structure: Title page, Table of contents, Introduction, concept/s description, Explanation of real life example, Association of concept to example, Conclusion
- The real life situation can be based on national newspapers (e.g. TOI, HT, Hindu) or national magazines (e.g. Business Today, Business India, PeopleMatters) available in the library or online

1. BIS - Low

The faculty has not provided any clarity on the assignment with regard to the following:

- The format of the document in terms of layout, font type and size and line spacing
- The structure and flow of the document
- The possible sources of information for the real life situation or phenomenon

**Climate of Trust (COT)**

1. COT - High

Your faculty has created an environment in which you find it easy to approach them as well as your fellow classmates for exchange of ideas and concerns about the assignment. You can depend upon faculty and classmates for giving you time, listening to your point of view and providing an honest feedback. You know for sure that ideas you have shared with others will not be used by them for their gain. The students believe that discussion with classmates amongst them can help improve the quality of their assignment. They have regard for each other’s opinions and perspectives, and are comfortable about seeking and providing clarifications.

1. COT - Low

Your faculty has created an environment in which you find it difficult to approach them as well as your fellow classmates for exchange of ideas and concerns about the assignment. You cannot depend upon faculty and classmates for giving you time, listening to your point of view and providing an honest feedback. You doubt the intention of others, and fear that yours ideas will be used by others for their gain if you share it with them. The students believe that discussion with classmates is futile and serves no purpose towards helping them improve the quality of their assignment. They have no regard for each other’s opinions and perspectives, and are uncomfortable about seeking and providing clarifications.

**APPENDIX B**

**SUMMARY OF STUDIES**

| **STUDIES** | **Study 1** | **Study 2A, 2B, 2C** | **Study 3** |
| --- | --- | --- | --- |
| **Objective** | To define and validate item scales for DMD, BIS and COT and the experimental vignettes for each | To confirm the impact of DMD, BIS and COT  on Thriving | To explore the relation of DMD, BIS and COT, Thriving and Self-Determination Components |
| **Research Method** | Experimental Study  2 (high vs low DMD)*  2 (high vs low BIS)*  2 (high vs low COT)  Between subjects design | A set of experimental vignette studies  2A) High vs low DMD  2B) High vs low BIS and  2C) High vs low COT  Between subjects design | Field Study |
| **Sample size** | N = 240 | N_2A_ = 60, N_2B_ = 60, N_2C_ = 60 | N = 92 |
| **Analysis** | Convergent and Discriminant Validity, Reliability  Manipulation Check | Manipulation Check  T-Tests | Hierarchical Regression |
| **Finding** | The item scales for DMD, BIS and COT  are valid and reliable | DMD, BIS and COT  impact Thriving | DMD, BIS and COT  increase Thriving  Competence partially mediates the relation between DMD and Thriving  Competence fully mediates the relation between BIS and Thriving &  COT and Thriving |

DMD: Decision Making Discretion

BIS: Broad Information Sharing

COT: Climate of Trust
